# Comparing patient and provider perspectives on a rural, multilevel, community-engaged Food is Medicine intervention

**DOI:** 10.1093/haschl/qxag072

**Published:** 2026-05-11

**Authors:** Danielle M Krobath, Vanessa Nicholson-Robinson, Yarisbel Melo Herrera, Kenneth Chui, Sujata Dixit-Joshi, Fang Fang Zhang, Cassandra Hawkins, Erin Hennessy, Lawren Long, Julian D Miller, Anastassios G Pittas, Daniel J Schultz, Christina D Economos

**Affiliations:** Department of Epidemiology and Biostatistics, Arnold School of Public Health, University of South Carolina, Columbia, SC, United States; Friedman School of Nutrition Science and Policy, Tufts University, Boston, MA, United States; Department of Public Health and Community Medicine, Tufts University School of Medicine, Boston, MA, United States; Friedman School of Nutrition Science and Policy, Tufts University, Boston, MA, United States; Department of Public Health and Community Medicine, Tufts University School of Medicine, Boston, MA, United States; Friedman School of Nutrition Science and Policy, Tufts University, Boston, MA, United States; Friedman School of Nutrition Science and Policy, Tufts University, Boston, MA, United States; Jackson State University, Jackson, MS, United States; Friedman School of Nutrition Science and Policy, Tufts University, Boston, MA, United States; Department of Social Science/Political Science, Tougaloo College, Jackson, MS, United States; Reuben V. Anderson Institute, Tougaloo College, Jackson, MS, United States; Division of Endocrinology, Diabetes and Metabolism, Tufts Medical Center, Boston, MA, United States; Friedman School of Nutrition Science and Policy, Tufts University, Boston, MA, United States; Friedman School of Nutrition Science and Policy, Tufts University, Boston, MA, United States

**Keywords:** food is medicine, produce prescription programs, rural health services, community-based participatory research, qualitative research, patient-centered care, healthcare provider attitudes, social determinants of health, food insecurity, health equity, cardiometabolic diseases, chronic disease prevention, program implementation, stakeholder engagement, healthcare related frustrations

## Abstract

**Introduction:**

Community engagement and multistakeholder alignment are critical for implementing effective Food is Medicine (FIM) programs. Together with the Delta Health Center and regional farmers in the Mississippi Delta, the Delta GREENS (Growing a Resilient, Enriching, Equitable, Nourishing food System) FIM Project is testing the effects of a multilevel, community-engaged intervention that provides weekly locally-grown produce boxes over 12 months on adult cardiometabolic outcomes.

**Methods:**

We conducted 3 focus groups with low-income patients with chronic disease risk (*N* = 25) and 2 with healthcare providers (*N* = 19) across 3 counties during formative stages of Delta GREENS. We compared patients’ and healthcare providers’ perspectives on potential impacts of FIM and logistical, context-specific implementation considerations, and thematically analyzed transcripts using an inductive approach.

**Results:**

Patients emphasized macro-level structural benefits (ie, increasing household purchasing power, boosting local economies) while providers emphasized individual-level benefits (ie, nutrition education) of Delta GREENS. Patients wanted box quantities scaled to household sizes, while providers voiced concerns about healthcare-related frustrations, such as patient non-adherence and attrition. Both groups agreed that produce boxes could support healthy eating.

**Conclusion:**

This study provides critical insights to better align stakeholder priorities to guide the design of culturally responsive FIM programs.

## Introduction

Food is Medicine (FIM) programs integrate food-based nutritional interventions into healthcare to treat or prevent disease.^1^ They involve a spectrum of programs, services, and other interventions. For instance, lower levels of the spectrum center population-level prevention of disease through broad policies and programs; whereas higher level interventions are medical treatment programs that target patient groups with specific medical conditions as well as social needs. FIM programs aim to improve healthy food access by shifting the cost of FIM programs to all types of health insurers. Recent modeling studies estimate that if Medicare, Medicaid, and private insurers implemented medically tailored meals for all patients with both a diet-related condition and the limited ability to perform daily activities, nearly $13.6 billion in health care spending could be reduced in 1 year (accounting for the cost of program implementation).^2^ While this strategy offers enormous potential benefits, such as reducing the economic burden of expensive healthy food costs on low-income patients, it relies on a “one-size-fits-all” model that prioritizes efficiency and cost-savings over cultural relevance, lived experiences, and structural drivers of health.^3,4^

In the United States, momentum is rapidly building around FIM as a viable tool to address food insecurity and the rising burden of preventable diet-related diseases, such as obesity, hypertension, and type 2 diabetes.^5^ Driven by the 2022 White House Conference National Agenda, FIM has been recognized as a key nutrition strategy to prevent chronic disease in the form of produce prescription programs, medically tailed meals and teaching kitchens as examples. FIM has gained recognition as a potential gateway of supporting the practice of equitable science methods designed to support interventions to reduce racial, ethnic, and socioeconomic disparities in diet-related diseases. FIM is defined as “a set of programs or services that aim to link food, nutrition, and health into health care systems.”^6^ Examples of FIM programs include medically tailored meals and “prescriptions” for healthy foods, such as free produce boxes or vouchers for produce redeemable at local farmers’ markets or grocery stores. Equitable science methods, such as prioritizing community-based participatory research that center cultural considerations and inclusivity, as well as multi-level evaluation, may be practiced in FIM programs to ensure nutritional interventions are effective, consistently accessible, and respectful of all communities, including those most impacted by diet-related diseases and historical disinvestment. FIM programs have been linked with significantly lowering food insecurity, increasing fruit and vegetable (FV) consumption, and improving cardiometabolic markers, such as body mass index (BMI) and hemoglobin A1c (HbA1c).^7,8^

Prior literature highlights important discrepancies between provider-oriented goals of FIM interventions and patients’ lived experiences of how nutrition guidance is delivered and accessed, raising questions about feasibility and implementation in real-world settings. Ridberg et al. (2025) conducted one of the first nationally-representative surveys of US adults’ knowledge, perceptions, and experiences regarding FIM.^6^ While most respondents strongly supported FIM, many reported not receiving consistent, or any, nutrition advice from their healthcare providers, highlighting a gap in even the most basic integration of nutrition into clinical care. The lack of attention on nutrition during medical encounters was most pronounced among low-income and uninsured populations, underscoring that significant progress is needed to integrate FIM and nutrition education into primary and preventative care nationwide. There is a need for studies that directly examine both provider and patient viewpoints to better understand gaps in FIM delivery and to inform more effective integration of nutrition care into primary and preventive healthcare.

FIM interventions have the potential to address health disparities, but doing so requires centering community voices and designing study components that align with the priorities and experiences of both patients and their healthcare providers, critical partners for ensuring program success and optimal health for all. Centering community voice is important for overall healthcare landscape and may be uniquely applied in FIM initiatives. For instance, previous qualitative studies show that when implementing FIM into clinical settings, healthcare staff prioritize cultural responsiveness and clearly delineating roles and expectations.^2,9^ Further, these expectations should be integrated into existing clinical workflows and electronic health record (EHR) systems. Qualitative research on patient priorities highlights the importance of personalized nutrition education, adjusting food amounts to accommodate household and family size, and building culturally appropriate cooking skills.^10^ However, few studies have focused on investigating how perspectives may align or differ across patients and healthcare providers, particularly in places with unique food and health challenges, such as the rural South.

Our study examines the variability between provider and patient perspectives, including their healthcare-related frustrations (HRF), food access, and health challenges, as well as solutions to address them via FIM programming. Such insight is necessary to design and implement place-based interventions that are effective and sustainable. Thus, the purpose of this qualitative study was to compare patients’ and healthcare providers’ perspectives on potential impacts of FIM and logistical, context-specific considerations for FIM programming. Insights were collected to design and implement The Delta GREENS (Growing a Resilient, Enriching, Equitable, Nourishing food System) FIM Project.

## Methods

### Design of the Delta GREENS FIM project

This qualitative study was part of the formative work conducted to inform the design of the Delta GREENS FIM Project (ClinicalTrials.gov #NCT06358859). Briefly, Delta GREENS is a 5-year multi-sectoral, community-engaged intervention in partnership with Delta Health Center (DHC) and local farmers in the Bolivar, Sunflower, and Washington counties of the Mississippi Delta. Using a randomized-controlled trial design, the intervention aims to improve cardiometabolic outcomes (eg, BMI, HbA1c) by providing fully subsidized fruit and vegetable boxes, primarily grown by local area farms, for 12 months, along with nutrition education and recipe cards. The formative work, described herein, utilized a community-engaged participatory research approach to inform the co-design of the intervention. Findings shaped the specific components and strategies implemented during the implementation stage of the Delta GREENS intervention. This work included the establishment of academic-community partnerships, community mapping and listening sessions, farm site visits, and focus groups. This manuscript describes findings from focus groups with DHC patients and health care providers.

### Study population

Focus groups were conducted with patients and health care providers at community sites in selected communities.

#### Providers

Providers were recruited using convenience sampling methods with our study partners at the sites where the intervention would be implemented. Eligibility criteria for the provider focus groups included being a DHC healthcare practitioner or staff actively employed as a medical doctor, physician assistant, physical therapist, family nurse practitioner or patient-facing staff (ie, nutritionist, health and wellness center staff).

#### Patients

The research team partnered with providers of the DHC to assist with patient recruitment using convenience sampling allowing patients to enroll on a first come first serve basis. Patient focus group eligibility included adults 21 years or older who were receiving healthcare services at 1 of the 6 DHC clinics. Additionally, focus group participants had to (1) reside in 1 of the 3 rural target counties (Bolivar, Washington, or Sunflower); (2) have a diagnosis of diabetes or high blood pressure within their households (ie, themselves or someone else); or (3) be classified as food insecure. These counties were chosen as sites of recruitment as (1) these counties currently house the various locations of the DHC facilities and (2) many DHC patients fit the study criteria for eligible enrollment.

### Recruitment

From June to August 2023, we recruited participants using multiple methods. Patients were recruited using flyers with a QR code posted at the 6 DHC clinics that connected them with study staff. Healthcare providers were recruited on a first-come, first-served basis through an email sent to their work email addresses by the Tufts Univeristy research team, using a list provided by DHC management. All interested individuals completed a self-administered screening questionnaire, and, if eligible, were prompted to (1) consent to participate in the study, (2) complete a brief sociodemographic questionnaire, and (3) indicate preferred communication methods (email or phone).

All focus group participants (patients and providers) were informed that participation was voluntary, would not influence their employment or receipt of services, and that responses would be kept anonymous and not shared with their employers or providers. Upon completing the focus groups, all participants received a $75 gift card and dinner for their time. All study procedures and materials were approved by the Tufts University Health Sciences Institutional Review Board (#00003781).

### Focus groups

Trained qualitative researchers (DS, CH and KMJ) conducted the five 60-minute focus groups using semi-structured guides.

Patients: Questions explored participants’ attitudes, behaviors, and barriers related to FV consumption and program participation. Patient focus groups were conducted in person at community sites. Nutrition knowledge was assessed by asking patients the following question: Are there any health benefits from eating fruits and vegetables? We also assessed the following domains:

#### Perceptions of fruit and vegetable intake

Importance of consuming fruits and vegetables assessed using Likert-scale ratings.

#### Purchasing and consumption behaviors

Recent grocery shopping patterns, fruit and vegetable purchases, types purchased, household availability (fresh, frozen, canned), and typical consumption timelines.

#### Food access and purchasing locations

Usual sources for fruits and vegetables, frequency of purchase, and perceived access at the individual and community levels.

#### Local food sources

Experience purchasing from local farmers, frequency and recency of purchases, perceived benefits, and barriers to purchasing locally.

#### Barriers to access

Individual, community, and store-related barriers to obtaining fruits and vegetables.

#### Produce distribution program feasibility

Willingness and ability to pick up weekly produce boxes, preferred pickup locations, interest in home delivery, and anticipated delivery-related challenges.

#### Program design preferences

Desired quantity of produce, responses to excess or insufficient produce, impact on personal purchasing behavior, and preferences for specific fruits and vegetables.

#### Program interest and participation

Interest in program participation, motivations, information needs, perceived barriers, and factors influencing successful engagement.

Healthcare Provider (Practitioners and Clinical Staff) Questions were designed to explore perspectives around implementing Delta GREENS, seeking input on the study's relevance, potential benefits and drawbacks, and target population. Focus groups with providers were conducted virtually using Zoom. The following domains summarize questions asked of healthcare provider:

#### Perceived need and value of the program

Providers’ views on the need for the program at DHC, perceived advantages and disadvantages relative to existing programs, and patient populations most likely to benefit.

#### Social determinants of health

Perceived impact of social determinants of health on patient participation and benefit, and strategies to mitigate these barriers.

#### Provider willingness and feasibility

Willingness to participate in the program, perceived benefits and concerns, barriers to participation, and factors influencing feasibility for both providers and patients.

#### Program fit with clinical workflow

Perspectives on how the program could be structured to align with DHC workflows and integrate into routine care.

#### Patient-centered program design

Recommendations for structuring the program to meet patient needs, promote enrollment among those most in need, and anticipated patient perceptions of the program.

#### Nutrition education components

Suggested nutrition education topics and preferred formats to support patient engagement and program effectiveness.

#### Implementation readiness

Confidence in implementing the program at DHC, anticipated implementation challenges, organizational characteristics affecting reach, and strategies to address barriers.

#### Referral processes

Confidence in identifying and referring eligible patients, and factors that may facilitate or hinder referral.

#### Evaluation and follow-up feasibility

Perceived challenges and facilitators related to patients returning for in-person measurements.

### Data analysis

Focus group sessions were audio-recorded and transcribed verbatim. A thematic analysis of qualitative data was conducted using an inductive coding approach where the codes and themes were established based off the feedback of the providers and patient population that was collected from their interviews. We chose this type of coding to avoid assumptions and bias, thereby allowing themes to be directly expressed from the community. DMK, VNR, and YMH, trained qualitative researchers, developed codes and operational definitions using evaluation, magnitude, and descriptive coding techniques. Codes were iteratively modified until consensus was reached. DMK and YMH coded all interviews using NVivo 14 (Lumivero, Melbourne, Australia) and held weekly meetings to discuss coding and agreement. NVivo's calculated Cohen's Kappa for each code was inspected to guide reflexive and interpretative dialogue between coders to reach consensus relating to coding decisions. DMK, VNR, YMH, and CDE collaboratively derived final themes and subthemes using focused and axial coding techniques.

## Results

A total of 29 patients and 19 providers participated in focus group discussions (3 focus groups with patients and 2 focus groups with providers). Most patients identified as women (*n* = 16, 55%), Black (*n* = 28, 97%), and rated their health as “Fair” (*n* = 14, 48%) or good (*n* = 10, 34%) (Table 1). Most participants reported either themselves or someone within their households had a diabetes (*n* = 27, 93%) or hypertension (*n* = 28, 97% diagnosis), and 17 (59%) indicated that they had enough food to eat but it was not their desired types. Among providers, most were women (*n* = 15, 79%), Black (*n* = 12, 63%), and physicians (*n* = 4, 21%) (Table 2).

**Table 1.** Sociodemographic characteristics of Delta health center patients (*N* = 29).

	*N* (%)^b^
**County of residence**	
County A	16 (55)
County B	5 (17)
County C	8 (28)
**Diabetes diagnosis (self or anyone in household)**	
Yes	27 (93)
No	2 (7)
**High blood pressure diagnosis (self or anyone in household)**	
Yes	28 (97)
No	1 (3)
**Food sufficiency (last month)** ^a^	
Enough of the kinds of foods wanted	8 (28)
Enough, but not always kinds of foods wanted	17 (59)
Sometimes not enough to eat	3 (10)
Often not enough to eat	1 (3)
**Self-rated health status**	
Good	10 (34)
Fair	14 (48)
Poor	5 (17)
**Gender**	
Female	16 (55)
Male	13 (45)
**Race**	
Black or African American	28 (97)
White	1 (3)
**Household size**	
1	5 (17)
2	11 (38)
3	5 (17)
4+	6 (21)
N/A (data not reported)	2 (7)

Source: Primary data collected by the authors.

^a^U.S. Department of Agriculture Food Sufficiency Screener.

^b^Percentages may not add up to 100 due to rounding.

**Table 2.** Sociodemographic characteristics of Delta health center healthcare providers (*n* = 19).

	*N* (%)
**Gender**	
Female	15 (79)
Male	2 (11)
N/A	2 (11)
**Race**	
Black or African American	12 (63)
White	3 (16)
Other race	2 (11)
N/A	2 (11)
**Role at DHC**	
Clinical psychologist	1 (5)
Healthcare administrator	2 (11)
Medical doctor (MD)	4 (21)
Nurse	4 (21)
Nurse practitioner or family nurse practitioner	4 (21)
Nutritionist	1 (5)
Pharmacist	1 (5)
Physician assistant	1 (5)
Physical therapist	1 (5)

Source: Primary data collected by the authors.

While prior findings have emerged from these data relating to how historical and structural barriers impact residents in these Delta communities,^11^ 6 additional themes were identified in this study. Three concerned the benefits of the intervention and potential mechanisms for influencing individual and community health (Topic 1, Table 3), and 3 concerned the logistical considerations for implementation, participant retention, and long-term sustainability (Topic 2, Table 4).

**Table 3.** Perceived benefits of the Delta GREENS intervention.

Theme 1. The Delta GREENS Intervention Will Facilitate Health Status Improvements (*Shared theme*)			
	Population	Selected patient quote(s)	Selected provider quote(s)
**Subtheme 1.1.** FV boxes will improve the health of recipients and their families through greater consumption of FV.	P HCP	“*It's a personal value. The decommodification of food is something that I actually stand for and value. I lost my mother to Type I diabetes. This does hit home in that regard. I would like to see genuine, uplifting, change because food ought to be a human right, in my honest opinion.”*	*“You know, here in the Mississippi Delta, we eat so much processed food and not enough fruit. These boxes, I think that, overall, the people will really benefit from it. They will come by and pick it up and it will help the outlying issues, such as we are always eating too much of this and not enough of that.”*
**Subtheme 1.2.** Providing free FV will promote consumption for low-income patients unable to afford fresh produce otherwise.	P HCP	“*Yes, quite frankly you're talking about the… predominantly African-American community and the ones who are on food stamps, for example, they may have five or six kids there. They have to go shop at… Walmart out in Cleveland. They can't afford to purchase fruit and vegetables because they got to feed those kids for the rest of the month. They have to get some rice and beans and stuff like this. Unfortunate, but that's what it is…. everybody talk that good talk about fruit and vegetables… ‘We should eat fruit.’ All of us love to eat fruit and vegetables all time, but we can't afford to eat fruit and vegetables. It's just that simple. We would love to, [and] when we get a chance, we do, but reality is what it is*.”	“*I think that it’ be a good idea since most of the patients that we have are in a low economic status. So, most of the patients are not able to control their diseases because they are not aware of what foods they are supposed to eat, or they cannot afford it. If there is something out there that will not only tell them what to eat but also provide the funds for them to get it, I think that would be perfect*.”

Theme 2. Delta GREENS may combat the structural determinants of food insecurity in the MS Delta (*Patient-only theme*)			
**Subtheme 2.1.** Intervention will alleviate the limited availability of fresh, high-quality produce.	P	“*[FIM] would help me out a lot because sometimes you get to the store, they ain't got what you want…. I went [to] every grocery store in Cleveland and they ain't got it…. Then you ask the people that's working in the store. “We don't have that.” You got to wait on a truck to come. People come in and buy so many [fruits and vegetables]. So, you still got to wait another week to wait on that truck.”* “*There's a minimal of fruits and vegetables in the Delta…. We don't have access with all the land that we have…. I've been in other places and you can find farmer's market and fruit markets everywhere. Here in the Delta where all this land, don't nobody grow anymore. I remember as a kid we used to have fig trees, plum trees. You can't even find a fruit tree in the yard no more. We don't have it. Then when you go in the grocery store, they look like crap. The fruits and the tomatoes are ruined. The lettuce, you have to stay longer to pick over to find some and it's not keeping good. I buy it and it's not holding well.”*	—
**Subtheme 2.2.** Intervention will alleviate transportation barriers and physical proximity to fresh, high-quality produce.	P	“*Quite frankly, financial covers a lot within itself. Not only this purchasing stuff but as we spoke about it earlier, the transportation to and from, we certainly don't have within our community, so we got to travel to another community to make these purchases. Obviously, there'd be a significant saving for each and every one of us to have fresh vegetables and fruit in one home and our community is something special.*” “*Basically, the concept of your local produce is oftentimes far from where your residency is. Say what we know as Southside, they have various little convenience stores, but it's not fresh produce. They have processed food and stuff like that, and that's a lack of accessibility, and there's no means to get access there.*”	—
**Subtheme 2.3.** Intervention will expand purchasing power, alleviating the effects of poverty.	P	“[*Free Fruit and Vegetable Boxes] would [save us] that additional gas money, and we also have to buy meat. You don't have to purchase vegetables, so you could save more [money].”*	—
**Subtheme 2.4.** Intervention will bolster the economy by supporting local farms and creating jobs.	P	“*[FIM] is probably making jobs too…we have more farmers to get people to work in their greenhouses.”*	—

Theme 3. Delta GREENS will improve individual determinants of diet-related disease management and healthcare delivery (*Provider-only theme*)			
**Subtheme 3.1.** Intervention will increase patients’ nutrition knowledge.	HCP	—	“*Telling [patients] what foods help with their condition, let them know, hey, eat more of this, it's better with fiber. This helps with this condition. eat less of this due to inflammation, so in providing them with it, and besides just saying, go buy it, we'll make them understand and make them try it.”* “*We're thinking about how to have that accompany education and support for our families as they are getting the vegetables and produce, how you know the best…best ways to kind of take advantage of that in terms of recipes. I know there's all kinds of ideas about that, and how to incorporate those amazing foods accordingly. I think for a lot of folks it's not just the accessibility, but also, that other piece, on how to incorporate those [foods] when you haven't had that exposure [to the foods].”*
**Subtheme 3.2.** Intervention will improve diet quality by exposing patients to new FV.	HCP	—	“*Introducing the right food, it will make it easier…. The box will introduce them to another variety of food that they're not used to in bringing down their A1C…because now they're getting something healthier than what they were eating*.”
**Subtheme 3.3.** Streamlined workflows for patient-provider communication	HCP	—	“*It's all about communication, reaching out to the patient, contacting the patient. Make sure we got the correct phone number, make sure we got correct address mailing, sending out text messages, email. It's all about proper communication in terms of getting the information out to the patients.*” “*Because I know some of our patients, a lot of our patients have access to technology, and you know, video and Internet, but not all of them do.*” “*Designating a person for the [FIM] programs to help them stay on top of things like, instead of just having the program out and just passing the boxes out all randomly like, have only keeping up with who gave them the box, a good phone number, someone that lives with the patient who can help them, as well as get them together in the clinic to help them help the patient. I think someone is constantly checking on the patient what would be best.”*

Source: Primary data collected by the authors.

**Table 4.** Potential barriers to sustained FIM programming in the Mississippi Delta.

Theme 4. Logistical considerations of Delta GREENS Program Delivery (*Shared theme*)			
	Population	Selected patient quote(s)	Selected provider quote(s)
**Subtheme 4.1.** Inconvenient delivery method of produce boxes.	P HCP	“*I'm going to say, for me because I don't drive. I don't have no money to pay nobody to rent a vehicle, nothing. I have to come by transportation*.”	“*I think accessibility in the clinic would definitely make it a whole lot easier. Not just produce half-life and having to worry about that. But just having them here, I think that puts it on our minds as providers to give it to patients, and also patients will ask for it as well if they see it, so that makes it more likely for us to give it out and make it accessible to our patients.”*
**Subtheme 4.2.** Misalignment of quantity of produce in boxes with household size.	P HCP	“*It's like you don't know how much anybody*—*You got children at home then that box ain't going to last the whole week. Like me, I got grandchildren at home, and I have to cook for them because they have to [feed] everybody in that cupboard for me and for three or four children.”* “*I would want to know if you know the box [quantities]… [we are] only two, me and my friend. Now, all that I don't eat I'm not going to let it go to waste. I'm going to share some of that stuff with some of my neighbors… so it don't go to waste because I don't like throwing food away”*	“*If it's enough to feed their family like, say, making sure it's enough, because many of them have big families. If it's enough to where they can make a meal from this or if you know, still got to go buy something to add to it to make a meal.”*
**Subtheme 4.3.** Compromised quality of produce.	P	“*For some reason we in Mississippi Delta, which is one of the poorest regions in the country. You're aware of that. Don't confuse poorness with uneducated and ignorance. What I mean clearly about that is for some reason people up north and other places, they want to make a contribution to the Delta in the way of fruit and vegetables. They send these little apples…Don't do that. Let us have decent fruit and vegetables. Don't give us something nobody else wants*.” “*A lot of foods are genetically engineered for desired results or just, better robustness and various different temperatures. Some people don't necessarily want the genetic engineering because of various different reasons. I don't necessarily care….but I am more concerned about the conventional means. A lot of these genetic engineered foods are made resistant to a lot of the chemicals that they use to help produce the food, pesticides and whatnot. I'm just curious, how clean is the produce that's coming to the door? Is it hard conventional means or is it organic means?…Honesty is key….I would just like that upfront, know how my food was handled.*”	—

Theme 5. Determinants of Patient Engagement and Adherence. (*Provider-only theme)*			
**Subtheme 5.1.** Compliance challenges	HCP	—	“*The only thing that I can think of is getting the patients to stay consistent. There is a compliance issue. They're not really compliant with everything they should be, no matter how [much] help you try to give them. Some of them just cannot comply, or they just don't want to do it. I think the best way to introduce the program would be to help them focus on the positive of it, like how is they really going to help them? It's just got to be really drilled into them that this is here to help them. A lot of patients that we have are really not compliant. Don't want to take the medicine, they don't want to eat the foods, they don't want to exercise, don't want to do anything. That'll be the only disadvantage I can think of is to keep them consistent, with the programs consistently*.”
**Subtheme 5.2.** Non-random recruitment and enrollment of patients	HCP	—	“*I'll be eager to participate in this [FIM] program…. We have a referral only] extension program that is free for all of our patients to have access to the wellness center, which is a fitness center gym. So those that come here, they've made that next step, they are committed to working on losing weight, committed to working on their A1C, committed to working on their hypertension, getting off meds or whatever their health goal is, or just continuing to be able to move, whether it's their arthritis in their joints or whether they've just been released from physical therapy, they’re working on a goal already being physically active. Now, we can add the part of eating healthier, because, of course, it's a lot when you come from doing neither one of those and you're trying to say, eat healthy and exercise. My patients are doing one step, and now we are adding the food on to it.”*
**Subtheme 5.3.** Varying patient literacy levels	HCP	*—*	“*I know some of my patients have very limited reading skills, so if you're printing up a recipe, they may or may not know what that means, may or may not have the tools to measure things, or to prepare them.”* “*In regard to the recipe cards, to try to just keep it simple so that people don't get overwhelmed, and also maybe add pictures on the recipe cards. I think that visual would really be good and not just a huge book of recipes, like keep it very simple, especially to start with, and they can. you know, do it easily, and not have too much information all at once.”*

Source: Primary data collected by the authors.

### Topic 1: potential benefits of Delta GREENS

#### Shared themes

Patients and providers agreed that produce boxes could support health and disease management by improving FV intake among participants (sub-theme 1.1). Alignment also emerged in the belief that providing FV at no cost is a strong mechanism for program success, as most patients are low-income and often cannot afford fresh produce (sub-theme 1.2) (Table 3).

However, diverging perspectives emerged regarding the specific ways in which the intervention may support health, as detailed in patient-only and provider-only themes below.

#### Patients-only themes

Patients highlighted that free produce boxes would help ameliorate the structural determinants of food insecurity through several mechanisms (theme 2). They noted that produce boxes would increase household availability of FV, as local stores often do not carry these items or the available stock is low-quality, or “rotten” and “spoiled” (sub-theme 2.1). Furthermore, because DHC clinics are centrally located and, for some patients, closer than grocery stores and supermarkets, patients reported that receiving free FV boxes that they could pick up at DHC clinics would help them overcome transportation barriers (sub-theme 2.2). Moreover, patients felt that Delta GREENS would bolster household purchasing power for other basic needs, such as gas and more food (sub-theme 2.3). Lastly, patients endorsed FIM programming as a way to strengthen the local economy, noting that purchasing produce from nearby farms could generate jobs in their communities (sub-theme 2.4).

#### Provider-only themes

Providers focused on the individual level determinants of diet-related disease management and healthcare delivery that would be improved by the intervention (theme 3). In particular, they emphasized that the program improve patients’ nutritional knowledge (sub-theme 3.1) and increasing diet quality by exposing them to new FV that they would not otherwise purchase or even know about (sub-theme 3.2). Embedded in this theme was also the belief that the intervention could help streamline workflows for patient-provider communication (sub-theme 3.3), which would ultimately improve healthcare delivery at an individual level.

### Topic 2: potential barriers to FIM program success

#### Shared themes

Shared themes emerged regarding the logistical barriers for the success, scalability, and sustainability of FIM programs in the MS Delta (theme 4). Both groups emphasized the need for convenient delivery and/or pickup of produce boxes; convenience included both the location of where and timing of when boxes are received (sub-theme 4.1). For instance, both groups noted that having the community health center as a central pick-up location would support program access and availability, ultimately increasing study participation and retention. Moreover, both groups warned against providing produce in quantities that did not match the size of the patient's household, emphasizing that these individuals are often responsible for ensuring entire families have enough to eat. This theme also captured concerns around food waste, with patients indicating that they would not want to throw food away if too much was received (sub-theme 4.2).

#### Patient-only themes

Around potential barriers, patients focused on the quality of the produce received in boxes (theme 4.3), highlighting the need to receive FVs that have not been compromised (ie, spoiled) by the time they receive it. They used language that highlighted dignity and human rights, centering food quality as a central component of FIM programming. Included in the sub-theme of quality was patient concerns around production practices involved in growing the FV, emphasizing the need for local connections, minimal use of pesticides, and skepticism around the types of produce that could realistically be grown in their local communities. To address this concern, patients expressed a desire for disclosure of the farms where produce was grown, including production practices (ie, pesticide use) for each box received.

#### Provider-only themes

Providers noted that the success of the FIM program will depend on patient engagement and adherence to study protocols (theme 5). Providers were concerned about high levels of non-compliance observed in other patient interactions (eg, low medication or physical activity program compliance) (sub-theme 5.1). Therefore, as a potential strategy to overcome this concern, providers suggested prioritizing patients who have already demonstrated a commitment to improving their health, such as those enrolled in pre-existing lifestyle behavior modification programs. This shared belief among providers was the basis for sub-theme 5.2, which is non-random recruitment and enrollment of patients, or the disproportionate interest in enrolling participants who are already participating in lifestyle related programming. Finally, providers consistently pointed out that their patients have varying levels of literacy (sub-theme 5.3) that must be prioritized by study teams when designing all patient-facing materials, such as recipe cards and recruitment materials.

## Discussion

This formative research expands upon ongoing national support for fair and inclusive FIM programming with attention to systemic barriers to health that may impact patient engagement and the effectiveness of similar produce prescription programs.^11^ The goal of this research was to integrate insights from patients and healthcare professionals into the design and implementation of the Delta GREENS intervention. Assessing the perspectives of these groups revealed a strong shared belief that a multilevel FIM intervention in the Mississippi Delta has the potential to improve health by increasing fruit and vegetable consumption among a population with prevalent food insecurity. Both groups discussed the importance of prioritizing making delivery or pickup of FV boxes convenient and adjusting the quantity of FV based on household size.

While the agreement between patients and providers around potential dietary improvements from FIM and willingness to engage in programming is consistent with existing literature,^12-14^ our study identified significant differences in stakeholder perspectives regarding the underlying mechanisms through which FIM produce boxes may improve health. Patients emphasized that FIM had the potential to reduce structural barriers to accessing and consuming healthy foods, such as transportation and lack of availability, while providers emphasized the individual benefits of the intervention, such as increasing patients’ nutrition knowledge.

Divergent opinions also emerged when discussing logistical considerations for the success, scalability, and sustainability of FIM programming. Providers cautioned against patients’ lack of adherence to medical treatment and advice, which they believed could translate to an unwillingness to consume FV, even when provided for free. Yet, patients pointed to their current food environment as the main hindrance to following healthy eating patterns. In fact, most patients expressed a strong desire to participate and indicated some knowledge of the dietary patterns associated with lowering disease risk and progression. If patients are expected to maintain a healthy diet but lack consistent access to affordable and appealing FV, their health outcomes will remain suboptimal. These discrepancies point to broader structural imbalances that interact within social, economic, and food environments. Our findings align with published literature highlighting the disconnect between patients and providers and broader healthcare-related frustrations (HRF).^15^ HRF can occur when unfulfilled needs during medical encounters cause feelings of dissatisfaction and annoyance. Patients with chronic conditions who experience communication difficulties with providers may become overwhelmed and frustrated, consequently impeding trust in the healthcare system, developing an aversion to accessing care, and perpetuating non-compliance with medical recommendations. Providers also experience HRF, which manifests as challenges with care management, delayed communication with specialists, and services being inconsistently accessible to patients, resulting in unmet needs.^16^ In our study, many healthcare professionals focused on patient noncompliance and low literacy levels, potentially as a result of underlying and preexisitng HRF. Similar to our findings, other studies have found provider HRF when patients do not adhere to instructions after consultations, emblematic of widespread burnout among healthcare providers in the United States. Nonetheless, these diverging views must be addressed to prevent worsening healthcare delivery inconsistency and inefficiency, which could threaten the effectiveness of FIM interventions.

Going forward, FIM programs should use or establish validated frameworks that mitigate and prevent HRF and its implications. HRF bidirectionally impacts both patients and providers; thus, FIM programming should focus on enhancing health and well-being for providers and other actors in the healthcare system, as well as patients.^17^ Cafer and colleagues suggest achieving solutions by applying the Sustainable Livelihoods Approach and the Community Capitals Framework.^18^ These strategies emphasize enhancing system-level capacities, improving flexibility, and accounting for the wide variety of actors in healthcare systems. For instance, FIM interventions that address overlapping policies, systems, and environments within clinical care settings to respond to the needs of both providers and patients may be more impactful, overall.

Fair, inclusive, and equitable FIM programming should deliver solutions that are attainable for people and communities with the greatest need. Persistent beliefs that health behaviors, such as dietary intake, result solely from personal choice or knowledge gaps fail to recognize that these behaviors are partly shaped by policy priorities that restrict autonomy and sustain intergenerational poverty, upward mobility, and other forms of systemic oppression. For example, in Mississippi, healthy foods are more expensive in counties with disproportionately high rates of obesity, including our study counties.^19^ This may relate to the high density of convenience and corner stores, which tend to offer less variety and fewer high-nutrient options, while charging higher prices for healthy foods, as compared with full-service grocery stores and supermarkets.^20,21^ These systemic barriers support the data from patients in our study, who emphasized a lack of access to fresh, high-quality produce retailers.

Healthcare providers and clinical staff—key partners in the FIM movement—may unintentionally reinforce biases in their daily practice about who deserves access to and would benefit most from FIM programs and interventions. In our study, healthcare professionals’ focus on treatment adherence may stem, partly, from the lack of focus on social determinants of health (SDOH) in medical school and professional healthcare training.^22^ Consequently, potential threats to the viability, success, and external validity of FIM programming may emerge if clinical partners are more inclined to target specific patient populations (i.e., those enrolled in other behavior modification programs) for inclusion in trials such as Delta GREENS. The implications of such actions have received limited attention in the existing literature and related policy discussions. As the FIM space continues to grow, future research is needed to understand how patient selection bias influences enrollment, as well as the generalizability of FIM outcomes.

Overall, advocacy efforts focused on improving food access and strengthening knowledge systems are also needed. Such insight can be used to improve and refine the recruitment of both patients and providers into FIM interventions, which may include SDOH education campaigns tailored to local healthcare partners. Such actions will help avoid the creation of new but inaccessible knowledge systems. FIM advocates must know and recognize the unique SDOH across diverse communities that limit individuals’ access to nutritious diets during planning, implementation, and replication, even when healthy foods are provided for free. Researchers should address the critical gap in cultural and context-based FIM solutions by co-creating study materials with target community members,especially patients and healthcare providers, to develop effective interventions.^4^

Findings from our study have several implications for multiple stakeholders involved in Delta GREENS as well as similar produce prescription programs aiming to improve chronic health for high-risk communities in rural areas of the country. For instance, providers expressed that recipients oftentimes do not adhere to medical instructions, including healthy eating, while patients emphasized the challenges for not adhering to such instructions (i.e., lack of consistent access to healthy food, household food insecurity, and economic stress). These experiences indicate the need for solutions that not only prevent unhealthy eating, but also account for the systems and structures that lead to the undesired outcome, such as poor diet quality, so that solutions address both populations’ needs. Practitioners can also strengthen the alignment of solutions by being better equipped to address important SDOH, such as food insecurity. It may be useful for future programs to strengthen community capacity and resource development via community gardens, multisectoral partnerships, and establishing new food policies that protect vulnerable communities.

Our research team has worked closely with local farmers and leaders of the community in sharing resources and training local residents, farmers, and leaders to continue ongoing work so that once the program ends, the communities hold the knowledge and resources necessary to sustain impact. On an ongoing basis, Delta GREENS investigators also co-write grants with community partners to continue this work. The next steps are to conduct a landscape of food policies within the state to establish a call to action for those involved in policy making to solidify program sustainability and stability.

### Limitations

While our study has many strengths, it is not without limitations. Specifically, our scope is limited to the Mississippi Delta area and may lack generalizability. Nonetheless, recommendations may align with similar rural populations across the United States. Additionally, we used non-equivalent scripts for each distinct participant group (patients and providers), which may have influenced the thematic differences observed. Also, variation in question framing and depth of probing could have influenced the emergent themes; however, all interviewers received equal training to overcome this potential limitation. Finally, the number of focus groups conducted were predetermined by project timelines rather than saturation. Nevertheless, there were no unique codes or sub-themes emerging for 1 particular focus group, indicating that saturation was nevertheless achieved.

## Conclusion

To optimize health and programmatic success, FIM solutions must align with community needs and desires. For example, provided FV should support dietary requirements and participant preferences. FIM research and policy must acknowledge that even within the same intervention, priorities may vary across different stakeholder groups. Broader issues, such as improving local food economies, require macro-level solutions, like localized food policies and investments that address structural barriers (eg, unaffordable or absent grocery stores). As FIM gains national recognition as a tool to improve population health, researchers and policymakers should widely adopt community-based research approaches to understand community capacity and support program sustainability.^23^

## Supplementary Material

qxag072_Supplementary_Data
